# Transovarial Transmission of Dolichos Yellow Mosaic Virus by Its Vector, *Bemisia tabaci* Asia II 1

**DOI:** 10.3389/fmicb.2021.755155

**Published:** 2021-10-25

**Authors:** Amalendu Ghosh, Buddhadeb Roy, Aarthi Nekkanti, Amrita Das, Shri Dhar, Sunil Kumar Mukherjee

**Affiliations:** ^1^Insect Vector Laboratory, Advanced Centre for Plant Virology, Indian Agricultural Research Institute, New Delhi, India; ^2^Division of Plant Pathology, Indian Agricultural Research Institute, New Delhi, India; ^3^Division of Vegetable Science, Indian Agricultural Research Institute, New Delhi, India

**Keywords:** *Geminiviridae*, DoYMV, Silverleaf whitefly, vertical transmission, infected progeny, replication, virus-vector relationship 3

## Abstract

The cultivation of dolichos bean [*Lablab purpureus* (L.) Sweet] has been severely affected by dolichos yellow mosaic virus (DoYMV, *Begomovirus*) transmitted by whitefly, *Bemisia tabaci* (Hemiptera: Aleyrodidae). We tested the transovarial transmission of DoYMV in next-generation *B. tabaci* by PCR, real-time PCR, Southern blot hybridization, and biological transmission. The eggs, laid by DoYMV-exposed *B. tabaci*, carry the virus in a unique pattern. Only the eggs laid in between 3 and 6 days post virus acquisition by a parent *B. tabaci* were DoYMV positive. When tested individually in real-time PCR, around 31–53% of the eggs carried the virus. The presence of DoYMV in ovaries and F1 eggs was further substantiated by the hybridization of a Cy3-conjugated nucleic acid probe complementary to the viral strand of DoYMV. Viral DNA was also detected in F1 adults and F2 eggs. *B. tabaci* progenies carried not only the DoYMV DNA but were also infective. The F1 adults transmitted DoYMV to all tested plants and produced strong yellow mosaic symptoms. An increase in viral copies from egg to nymphal stage indicated propagation of DoYMV in *B. tabaci*. However, the increase was for a short period and decreased thereafter. The present study provides the first evidence of transovarial transmission and propagation of a bipartite begomovirus in its vector, *B. tabaci* Asia II 1. The transovarial transmission and replication of DoYMV in *B. tabaci* have great epidemiological relevance as *B. tabaci* can serve as a major host of the virus to bridge the gap between the cropping seasons.

## Introduction

*Begomovirus* (family *Geminiviridae*, order *Geplafuvirales*) is the largest known genus of plant viruses, which contains more than 445 virus species (ICTV, 2020). Begomoviruses are single-stranded (ss) circular DNA viruses that are either monopartite having single genomic DNA or bipartite with two DNA components viz. DNA-A and DNA-B (Brown et al., 2015; Krenz et al., 2015). Most of the begomoviruses are phloem limited and restricted to the vascular system except bean dwarf mosaic virus that invades mesophyll tissue (Levy and Czosnek, 2003). Infected plants show chlorosis, yellow mosaic, vein yellowing, vein thickening, leaf curling, flower bud abscission, and stunting. Begomoviruses have become widespread in South Asia and cause an estimated yield loss of 20–70% in beans, cassava, cotton, cucumber, melons, peppers, pumpkin, and tomatoes (Dasgupta et al., 2003).

Begomoviruses are transmitted by Silverleaf whitefly (*Bemisia tabaci* Gennadius, Aleyrodidae: Hemiptera). *B. tabaci* is considered a complex of morphologically indistinguishable cryptic species. To date, 46 such cryptic species are known (Rehman et al., 2021). Invasion of *B. tabaci* cryptic species MEAM1 and MED are thought to be responsible for outbreaks of several begomoviruses worldwide. The cryptic species of *B. tabaci* exhibit variations in host preference, insecticide resistance, and virus transmission (Alemandri et al., 2015). *B. tabaci* transmits begomoviruses in a persistent-circulative manner and thus the virions pass along the food canal after ingestion and reach the digestive tract. They are bound by the receptors located at midgut of *B. tabaci*. It is presumed that the virus crosses through the filter chamber to enter the hemolymph (Czosnek et al., 2017). The virus particles are circulated through hemolymph in coated vesicles and translocated into the primary salivary glands. The particles are egested with the saliva during feeding and salivation. Once *B. tabaci* becomes viruliferous, it remains so throughout its lifespan. Begomoviruses are not reported to multiply in their vectors except in the case of tomato yellow leaf curl virus (TYLCV). The amount of TYLCV DNA in *B. tabaci* increases initially and decreases thereafter (Ohnishi et al., 2009; Pakkianathan et al., 2015; Wang et al., 2016). However, it remains dubious as other studies concluded that there was no replication of TYLCV in *B. tabaci* (Becker et al., 2015; Sánchez-Campos et al., 2016). In general, begomovirus does not invade the reproductive organs of *B. tabaci* and the infectivity is not passed down to the progenies transovarially. The next-generation *B. tabaci* needs to acquire the virus again to become viruliferous. However, to date, there are at least two exceptions: monopartite begomoviruses TYLCV and tomato yellow leaf curl Sardinia virus (TYLCSV). TYLCV and TYLCSV have been reported to be transovarially transmitted by *B. tabaci* MEAM1 and MED (Ghanim et al., 1998; Bosco et al., 2004). We report here the transovarial transmission of a bipartite begomovirus, *Dolichos yellow mosaic virus* (DoYMV), by *B. tabaci* Asia II 1. During a screening program of dolichos germplasms, *B. tabaci* population was collected from the dolichos field and reared two generations on a non-host crop. A few young seedlings that were mock inoculated by *B. tabaci* showed characteristic yellow mosaic symptoms. The initial observations instigated us to investigate the transovarial transmission of DoYMV by its vector, *B. tabaci*.

DoYMV contains two similar-sized genomic components, DNA-A and DNA-B, each approximately 2.7 kb in size. The spread of DoYMV has affected the production of dolichos in South Asia (Capoor and Varma, 1950; Ramakrishnan et al., 1972; Swanson et al., 1992). Diseased plants produce faint chlorotic specks on leaves initially that develop into characteristic bright yellow mosaic patches. Early infections cause significant reductions in pod yield (Capoor and Varma, 1950). So far, mostly the RNA viruses such as reoviruses, rhabdoviruses, and tenuiviruses have been confirmed to be transmitted transovarially. A virus needs to either propagate or accumulate in sufficient amount to pass down the infectivity in the progenies (Mims, 1981). Besides, the virions must invade the reproductive system of the vector. Among the begomoviruses, monopartite TYLCSV DNA can be inherited in the progenies but is unable to give rise to infections, therefore lacks epidemiological relevance. TYLCV infection is transovarially transmitted only by *B. tabaci* MEAM1 but not by other cryptic species including *B. tabaci* Asia II 1 (Ghanim et al., 1998; Guo et al., 2019). The present study provides the first evidence of propagation and transovarial transmission of DoYMV infection by *B. tabaci* Asia II 1. The transovarially infected progenies reproduced the DoYMV infection in host plants. The vertical transmission of DoYMV by *B. tabaci* from parent to offspring in a propagative and transovarial manner has great relevance in virus epidemiology and management.

## Materials and Methods

### Virus Inoculum and Whitefly

The initial inoculum of DoYMV was collected from dolichos plants at the experimental field of Indian Agricultural Research Institute (IARI), New Delhi, India. The virus culture was established on dolichos plants (var. Pusa Garima) by *B. tabaci* inoculation and maintained under insect-proof conditions. The virus isolate was characterized based on the nucleotide sequence of DNA-A as described later.

An isofemale, virus-free line of *B. tabaci* Asia II 1 maintained for more than 5 years at Insect Vector Laboratory, Advanced Centre for Plant Virology, IARI was used in this study. The identity of *B. tabaci* was substantiated by sequencing mitochondrial cytochrome oxidase subunit I (mtCOI). Representative individuals of the population were randomly tested in PCR with generic begomovirus primers (Roy et al., 2015) to confirm its aviruliferous status. The freshly emerged adult females were collected using an aspirator for further experiments.

### DNA Extraction From *Bemisia tabaci* and Plant

Total DNA was extracted by using cetyltrimethylammonium bromide (CTAB) methods with modifications (Rehman et al., 2021). In brief, CTAB extraction buffer was prepared in a total volume of 1 ml containing 100 mM Tris–HCl (pH 8.0), 1.4 M NaCl, 20 mM EDTA (pH 8.0), 2% CTAB, and 2 μl β-mercaptoethanol. Individual *B. tabaci* was crushed in 100 μl of CTAB extraction buffer inside a microfuge tube using a homogenizer. About 100 mg of plant tissue was crushed in 500 μl of extraction buffer using mortar–pestle. The lysate was vortexed and incubated at 65°C for 30 min. An equal volume of chloroform/isoamyl alcohol (24:1) was added to the lysate and centrifuged at 16,000 × *g* for 15 min. The upper aqueous phase was transferred to a fresh microfuge tube. The DNA was precipitated by adding 0.7 vol of ice-cold isopropanol and kept at −20°C for 1 h. The sample was centrifuged at 16,000 × *g* for 15 min and the supernatant was decanted gently. The pellet if any was gently washed with 100 μl of 70% ethanol. The ethanol was decanted and residual ethanol was removed by drying at room temperature. The pellet was dissolved in 20 μl sterile distilled water in the case of *B. tabaci* and 50 μl in the case of the plant. The concentration and purity of DNA were assessed in a spectrophotometer (NanoDrop One; Thermo Fischer Scientific, United States).

### Characterization of *Bemisia tabaci* and Begomoviruses

A partial fragment of the mtCOI gene of *B. tabaci* was amplified using primer pairs C1-J-2195 and TL2-N-3014 (Simon et al., 1994). PCR was carried out in a 25-μl reaction mixture containing 12.5 μl of 2 × PCR Master Mix (Thermo Fischer Scientific), 0.4 μM of each forward and reverse primer, 8.5 μl of nuclease-free water, and 2 μl (∼50 ng) of DNA template. The PCR was performed in a T100 Thermal Cycler (Bio-Rad, United States) as described by Rehman et al. (2021) and resolved on an agarose gel stained with GoodView (BR Biochem, India). The purified PCR product was sequenced bi-directionally and sequences were processed by BioEdit and BLASTn. A consensus sequence was submitted in the GenBank. A Bayesian Inference (BI) phylogeny was undertaken using MrBayes 3.2 considering a genetic divergence cutoff of 4% to confirm the cryptic species identity.

Initially, begomovirus infection in the dolichos plants collected from the experimental field was confirmed by PCR with generic degenerate primers for begomoviruses, GEM-F and GEM-R targeting DNA-A (Roy et al., 2015). PCR-positive samples were subjected to rolling cycle amplification (RCA) to amplify the circular DNA of the begomoviruses. A 20-μl RCA reaction was prepared using 10 ng template DNA, 0.2 mM dNTPs, 7 U Phi29 DNA polymerase (Thermo Fischer Scientific), 2 × exo-resistant random primer (Thermo Fischer Scientific), 0.4 U pyrophosphatase (Thermo Fischer Scientific), and 1 × Phi29 DNA polymerase buffer. RCA was performed at 65°C in a water bath for 18 h. The RCA products were linearized by restriction digestion with EcoR1. The linearized RCA products were ligated into a pUC18 vector and transformed into DH5α *Escherichia coli* cells. Plasmid DNA was extracted using Wizard Plus SV Minipreps DNA Purification System (Promega, United States) and sequenced. The sequences were processed by BioEdit and BLASTn was performed to check the species homology.

### Standardization of DoYMV-Specific PCR and Real-Time PCR

A pair of DoMYV-specific primers (AG223F and AG224R, Table 1) was designed for the detection of DoYMV in PCR and real-time PCR. All the nucleotide sequences of DoYMV available in NCBI were aligned in ClustalW and primers were designed from the conserved regions in the coat protein (CP) using Primer 3 Input version 0.4.0. The major aspects such as primer length, amplicon size, GC contents, intra-primer, and inter-primer homology were considered for primer designing. The site specificity of the primers was checked in Primer-BLAST^1^. The newly designed primers were validated in a gradient PCR. Each 25-μl PCR reaction contained 12.5 μl of 2 × PCR Master Mix (Thermo Fischer Scientific), 0.4 μM of each forward and reverse primer, 8.5 μl of nuclease-free water, and 2 μl of DNA template (∼50 ng for *B. tabaci* and ∼100 ng for plants). The PCR was performed in a T100 Thermal Cycler with one cycle of initial denaturation at 95°C for 5 min, 30 cycles of denaturation at 95°C for 30 s, annealing at a temperature gradient of 50–55°C for 30 s, and extension at 72°C for 45 s followed by a final extension at 72°C for 10 min. The PCR products were resolved on 2% agarose gel stained with GoodView and visualized in a gel documentation system with a 100 bp plus DNA ladder. The annealing temperature that produced the best amplification without any secondary amplification was considered for detection of DoYMV in PCR and real-time PCR.

**TABLE 1:** List of primers and probe used in the study.

Target gene	Primer/probe name	Sequence (5’–3’)	References
*Bemisia tabaci* mtCOI	C1-J-2195-F	TTGATTTTTTGGTCATCCAGAAGT	Simon et al., 1994
	TL2N3014-R	TCCAATGCACTAATCTGCCATATTA	
Begomovirus DNA A	GEM-F	ATRRTHTGGATGGAYGARAACAT	Roy et al., 2015
	GEM-R	AAATCCCCTNTATTTCAAARAT	
DoYMV CP	AG223F	CCCGATATCGAATGCACGGA	This study
	AG224R	GTGGTTGTGAGGGACCATGT	
DoYMV CP	AG223F-Cy3 probe	Cy3-CCCGATATCGAATGCACGGA	This study

Real-time PCR was performed in a 48-well Insta Q48M (Himedia, India). A 20-μl reaction mixture consisted of a 10 μl 2 × DyNAmo ColorFlash SYBR Green qPCR Mix (Thermo Fischer Scientific), 2 μl template DNA (∼50 ng for *B. tabaci* and ∼100 ng for plant), and 0.5 μM of each forward and reverse primer. Thermal cycling was performed with one cycle of initial denaturation at 95°C for 5 min, 30 cycles of denaturation at 95°C for 30 s, annealing at 53°C for 30 s, and extension at 72°C for 45 s. Since SYBR Green I dye binds non-specifically to any double-stranded DNA, dissociation or melting stage was carried out after every reaction to determine the specificity of the amplification. Each of the biological replicates had three technical replicates. A no-template control (NTC) and positive control were included in each assay. The *C*_*T*_ values thus obtained were used to calculate the mean *C*_*T*_ and SEM using Microsoft Excel software.

### Acquisition of DoYMV by Adult *Bemisia tabaci*

Freshly emerged aviruliferous *B. tabaci* adult females were allowed to feed on a DoYMV-infected dolichos twig for 48 h of virus acquisition. Ten sets were maintained for virus acquisition, each containing around 50 adult flies. A few adults were randomly collected and tested in PCR as described previously to confirm the virus acquisition. The viruliferous adults were used to examine the transovarial transmission. All experiments were conducted in an insect-proof growth chamber at 27 ± 2°C, 60 ± 10% relative humidity, and 16 h light–8 h dark photoperiod.

### Detection of DoYMV DNA in Eggs and Next-Generation Adults

Viruliferous adult females of *B. tabaci* were collected in groups of 10 flies from each virus acquisition set. Each group of viruliferous flies was released on 4-leaf stage eggplants (var. Navkiran, Mahyco), non-host for DoYMV, and covered with an insect breeding box (SPL Life Sciences, Korea) with nets at two sides and top. All the sets were kept in an insect-proof growth chamber at 27 ± 2°C, 60 ± 10% relative humidity, and 16 h light–8 h dark for egg-laying. The adult flies were shifted to a fresh eggplant daily for up to 10 days.

To determine the pattern of transovarial transmission of DoYMV in next-generation (F1) eggs, the eggs laid by the viruliferous parent *B. tabaci* each day on eggplants were collected separately in 10 biological replicates. Total DNA was isolated from a group of eggs day-wise and tested in PCR with DoYMV-specific primers as described previously.

To assess the frequency of transovarial transmission of DoYMV in F1 eggs, approximately 10 eggs from each day laying were randomly collected using sterile needles. All the eggs were washed in 70% ethanol to eliminate surface contaminations if any. Total DNA was extracted from individual eggs and tested in real-time PCR for the presence of DoYMV as described previously. Ten biological replicates with three technical replicates were tested. The percent of eggs infected by DoYMV in each biological replicate was calculated as the number of eggs found positive out of 10 eggs tested in real-time PCR.

To determine the pattern of DoYMV infection in F1 adults, F1 eggs laid by viruliferous parents on eggplants were incubated at 27 ± 2°C, 60 ± 10% relative humidity, and 16 h light–8 h dark. The freshly emerged F1 adults were collected separately from each day laying. Total DNA was isolated from each set of adult flies and the presence of DoYMV was tested in PCR as described previously. Ten biological replicates of adult flies were tested. The eggplant on which eggs were laid and the adults were raised, was also tested in PCR to confirm the absence of DoYMV.

The proportion of F1 adults carrying the virus was estimated by randomly collecting 10 freshly emerged adult flies from each day laying. Total DNA was isolated from individual adults. The presence of DoYMV was detected in individual adults by real-time PCR as described previously with 10 biological and three technical replicates. The proportion of F1 adults carrying DoYMV DNA was expressed as a percentage. A *t*-test was performed to determine the significant difference among the mean proportion of eggs and flies carrying DoYMV. Mean differences among the categories were separated by Tukey’s test at a CI of 95% using XLSTAT 2014.5.03.

### Detection of DoYMV DNA in Second-Generation Eggs

Based on the results of detection of DoYMV from each day laying, a portion of F1 eggs laid in between 3 and 6 days post virus acquisition was used for generating next-generation adults (F1). The freshly emerged F1 adults were released on healthy eggplants (non-host) for egg-laying in five biological replicates. The second-generation eggs (F2) were collected from eggplants and tested in PCR for the presence of DoYMV as described previously. The host plant on which eggs were laid was also tested to confirm the absence of the virus.

### Transmission of DoYMV Infection by Next-Generation Adults

Based on the results of detection of DoYMV in F1 adults, the adults that emerged from eggs laid in between 3 and 6 days post virus acquisition were used for the biological transmission of DoYMV in dolichos plants. Healthy dolichos plants (var. Pusa Garima) were raised in an insect-proof plant growth chamber. All the plants were tested in PCR before DoYMV inoculation to confirm their healthy status. The F1 adults were released on healthy dolichos plants at a 4-leaf stage for 48 h of inoculation feeding. Fifteen plants were inoculated in three replicates. Ten adult flies per plant were used for inoculation. An equal number of plants were mock inoculated with aviruliferous *B. tabaci* that were not exposed to DoYMV. The inoculated plants were maintained under insect-proof conditions at 27 ± 2°C, 60 ± 10% relative humidity, and 16 h light–8 h dark photoperiod. All the plants were regularly monitored for symptom development and tested in PCR for infection of DoYMV at 15 days post inoculation.

### Localization of DoYMV in *Bemisia tabaci* Eggs

To understand the localization of DoYMV in *B. tabaci*, a tetramethylindo(di)-carbocyanine (Cy3)-conjugated fluore- scence oligonucleotide probe AG223F-Cy3 (Table 1) was used. The probe was conjugated with Cy3 at its 5’ end complementary to 20 nucleotide sequences in the viral strand of DoYMV CP. The invasion of DoYMV in the reproductive system and eggs of *B. tabaci* was localized using the Cy3-probe. The ovaries of DoYMV-exposed *B. tabaci* parents were dissected post 48 h of virus acquisition under a stereomicroscope. The eggs laid on 4-day post DoYMV acquisition by parent *B. tabaci* were used for DoYMV localization. The hybridization procedure was followed as described by Ghanim et al. (2009) with modifications. The specimens were washed several times with sterile water and fixed by using Carnoy’s fixative (chloroform/methanol/glacial acetic acid, 6:3:1, v/v) for 2 h at room temperature. The specimens were washed thrice with a hybridization buffer containing 20 mM Tris–HCl (pH 8.0), 0.9 M NaCl, 0.01% sodium dodecyl sulfate (SDS), and 30% urea. The hybridization was performed overnight with Cy3-probe at 10 pmol/ml in hybridization buffer. The specimens were washed three times with hybridization buffer and mounted on a glass slide. The Cy3-labeled specimens were observed under a confocal laser scanning microscope (TCS SP5 II; Leica, Germany). The photographs were captured and processed in LAS AF software (Leica). Ovaries and eggs of aviruliferous *B. tabaci* not exposed to DoYMV were taken as untreated control.

### Preparation of Standard Curve and Estimation of DoYMV Copies in Different Life Stages of *Bemisia tabaci*

For preparing a standard curve of DoYMV, the PCR-amplified product of DoYMV using primer pair AG223F and AG224R was ligated in a pJET1.2 vector using CloneJET PCR Cloning Kit (Thermo Fischer Scientific) and transformed into DH5α *E. coli* cells. A 10-fold serial dilution of the linearized plasmid DNA from 1 to 10^–5^ ng was used as a template in real-time PCR with three technical replicates. Real-time PCR was performed as described previously. Each reaction was followed by the dissociation or melting stage. The standard curve for DoYMV was prepared by plotting a linear regression curve with the mean *C*_*T*_ values on *Y*-axis and log DNA dilution (ng) on *X*-axis.

Virus titer present in eggs, third instar nymphs, freshly emerged adults, and 10-day-old adults of next-generation *B. tabaci* post virus acquisition by parents were assessed in real-time PCR. DNA was isolated from individual *B. tabaci* and used as a template in real-time PCR. Real-time PCR was performed with primer pair AG223F and AG224R as described previously. The absolute quantification of DoYMV present in individual *B. tabaci* egg, third instar nymph, freshly emerged, and 10-day-old adults was carried out by fitting the mean *C*_*T*_ values in the standard curve. DoYMV titer calculated in nanograms using the standard curve was converted into the copy number of DoYMV using the following formula. *N* = (X ng × 6.0221 × 10^23^ molecules/mole)/(*n* × 330 g/mol × 10^9^ ng/g), where *N* is the number of viral copies, *X* is the amount of amplicon (ng), and *n* is base pairs of recombinant plasmids. The average copy numbers of DoYMV were calculated along with standard error out of three biological and three technical replicates. ANOVA was used to test the significant difference among the virus copies in different life stages of *B. tabaci*. Mean differences among the categories were separated by Tukey’s test at a CI of 95% using XLSTAT 2014.5.03. The increase and decrease in DoYMV copies in different life stages of *B. tabaci* were compared to understand the virus propagation.

## Results

### Characterization of Whitefly and Virus

PCR with primer pair C1-J-2195 and L2-N-3014 produced an expected amplicon of ∼860 bp of *B. tabaci* mtCOI as visualized on 1% agarose gel (data not shown). The consensus nucleotide sequence exhibited about 99.99% identity with other *B. tabaci* sequences in NCBI upon BLASTn analysis. The sequence is available in GenBank with accession no. MT920041. In BI phylogeny with 4% genetic divergence cutoff, the present population clustered with other *B. tabaci* Asia II 1 cryptic species.

Linearization of the RCA product of begomovirus positive sample by EcoR1 digestion produced ∼2.7-kb amplicon on 1% agarose gel. The identity of the virus was confirmed by sequencing the cloned RCA product. Bidirectional sequencing of the cloned product produced 2,761 nt sequence comprising complete DNA-A that showed nearly 98.19% homology to other DoYMV isolates in BLASTn analysis. The sequence can be retrieved by GenBank accession no. MZ821026. The primer pair, AG223F and AG224R, targeting DoYMV CP produced ∼180 bp sharp band in gradient PCR at annealing temperature 53–55°C. Based on the quality of amplification, annealing temperature was standardized at 53°C for PCR and real-time PCR assays. The nucleotide sequences of the amplicon (GenBank accession no. MZ503593) showed 96% homology with other DoYMV isolates.

### Transovarial Transmission of DoYMV

**FIGURE 1:**
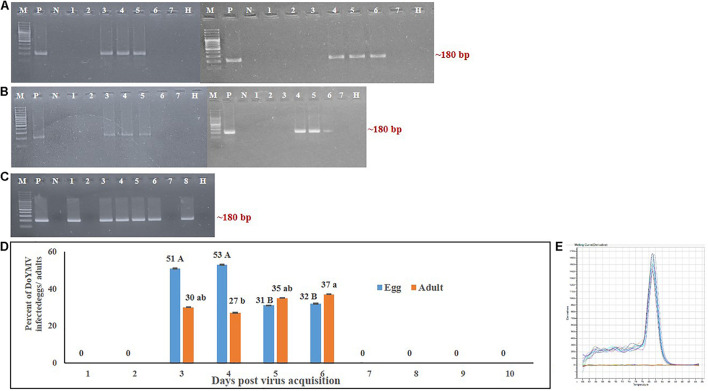
Detection of DoYMV in next-generation eggs and adults of *Bemisia tabaci.* **(A)** DoYMV was detected in eggs laid by viruliferous parents on different days post virus acquisition. DoYMV was detected in eggs 3–6 days post virus acquisition. **(B)** DoYMV was detected in F1 adults that emerged from eggs laid by viruliferous parents 3–6 days post virus acquisition. M: 100 bp plus DNA ladder, lane P: positive control, lane N: negative control, lanes 1–7: DoYMV-specific amplification from F1 eggs/adults laid by viruliferous parent 1–7 days post virus acquisition, lane H: non-host plant on which F1 eggs were laid and adults were raised. **(C)** Infection of DoYMV was detected in F2 eggs laid by DoYMV-positive F1 adults. A portion of F1 eggs laid between 3 and 6 days post virus acquisition was used for generating next-generation adults (F1). The second-generation eggs (F2) were collected and tested in PCR. Lane M: 100 bp plus DNA ladder; lane P: positive control; lane N: negative control; lanes 1–8: PCR amplicons of DoYMV from F2 eggs laid by F1 adults emerged from F1 eggs of parents 3 days (lanes 1, 2), 4 days (lanes 3, 4), 5 days (lanes 5, 6), and 6 days (lanes 7, 8) post virus acquisition; lane H: non-host plant on which F1 and F2 generations were raised. **(D)** Around 31–53% of F1 eggs laid in between 3 and 6 days post virus acquisition were DoYMV positive in real-time PCR. DoYMV was also detected in F1 adults (27–37%) that emerged from the eggs laid in between 3 and 6 days post virus acquisition. Data are means ± SEM of 10 biological and three technical replicates. 10 eggs and flies were tested from each day laying of each biological replicate, *n* = 100. Errors bars are SEM. Means denoted by different letters indicate a significant difference (*p* < 0.05). **(E)** Melt curves of DoYMV amplicons in real-time PCR. The specific melting temperature for DoYMV product was around 81°C.

The transovarial transmission of DoYMV was detected in PCR by testing a group of eggs laid at different days post virus acquisition. The eggs laid immediately after the virus exposure were free from DoYMV viral DNA. The first invasion of DoYMV in eggs was detected 3 days post virus acquisition (Figure 1). Eggs laid in between 3 and 6 days post virus acquisition were DoYMV positive in PCR. The presence of DoYMV DNA was not recorded beyond 6 days post acquisition. The virus titer in individual eggs beyond 6 days might either be too low to detect in PCR or not present at all. An increase in DoYMV exposure to *B. tabaci* parents extended the virus passage in F1 eggs beyond 6 days.

Ten randomly selected individual eggs laid by the DoYMV-exposed parents from each of 10 virus acquisition sets were tested in real-time PCR. The real-time PCR further substantiated the PCR results. No virus was detected in individual eggs before 3 days and after 7 days post DoYMV acquisition by parent *B. tabaci*. About 51% of the eggs laid on 3 days post virus acquisition were DoYMV positive in real-time PCR (Figure 1). An almost equivalent proportion (53%) of eggs laid on 4 days post acquisition carried DoYMV viral DNA. The proportion of DoYMV-positive eggs dropped slightly on 5 to 6 days post acquisition to 31%. A single specific peak at 81°C in melt curve analysis indicated specificity of the reactions.

A similar trend of DoYMV infection was recorded in F1 adults. F1 adults emerged from the eggs around 3 weeks post oviposition. Only the F1 adults generated from eggs that were laid by DoYMV-exposed parents in between 3 and 6 days post virus acquisition were DoYMV positive in PCR (Figure 1). F1 adults emerged from eggs laid before 3 days and after 7 days post virus acquisition by parent *B. tabaci* were free from DoYMV infection. About 27–30% of adults that emerged from eggs laid on 3–4 days post acquisition were found DoYMV positive in real-time PCR. The proportion of F1 adults with DoYMV DNA raised to 37% on 5–6 days post virus acquisition. A single peak specific to DoYMV in the melt curve analysis of real-time PCR indicated the specificity of reactions. DoYMV was not detected in F1 adults that emerged from eggs laid 7 days onward. A slight decrease in the proportion of virus infection in adults than eggs might be due to the fall of virus titer in individual adults beyond the detection limit of real-time PCR.

The F2 eggs laid by the DoYMV-positive F1 adults were also assessed to understand the passage of DoYMV from the first to second generation of *B. tabaci* progenies. F2 eggs laid by DoYMV-positive F1 adults retained the DoYMV viral DNA as detected in PCR (Figure 1). This was indicative of the passage of DoYMV beyond the first-generation progenies of *B. tabaci*. The eggplant on which the next-generation *B. tabaci* was reared, had been found DoYMV negative in PCR.

### Transmission of DoYMV Infectivity to Host Plants by F1 Adults

**FIGURE 2:**
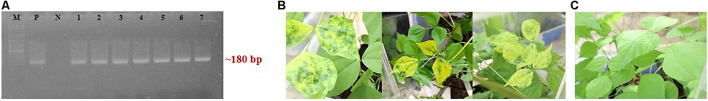
Infectivity of DoYMV-positive F1 adults of *B. tabaci*. Healthy dolichos plants were inoculated by F1 adults emerged from eggs laid between 3 and 6 days post virus acquisition by parent *B. tabaci*. **(A)** All the inoculated plants were found DoYMV positive in PCR at 15 days post inoculation. Lane M: 100 bp plus DNA ladder, lane P: positive control, lane N: negative control, lanes 1–7: test plants inoculated by F1 adults. **(B)** Inoculated plants developed characteristic bright yellow mosaic symptoms at 21 days post inoculation. **(C)** Mock-inoculated dolichos plants did not produce any symptoms.

To assess the infectivity of DoYMV-positive F1 adults, biological transmission was conducted. F1 adults emerged from eggs laid in between 3 and 6 days post virus acquisition by viruliferous parent *B. tabaci*, transmitted DoYMV successfully to healthy dolichos plants. All the test plants inoculated by F1 adults were found positive to DoYMV in PCR at 15 days post inoculation. The characteristic bright yellow mosaic symptoms appeared at 21–25 days post inoculation (Figure 2). Plants that were mock inoculated by aviruliferous *B. tabaci* were found PCR negative and did not produce any symptoms.

### Localization of DoYMV in *Bemisia tabaci*

**FIGURE 3:**
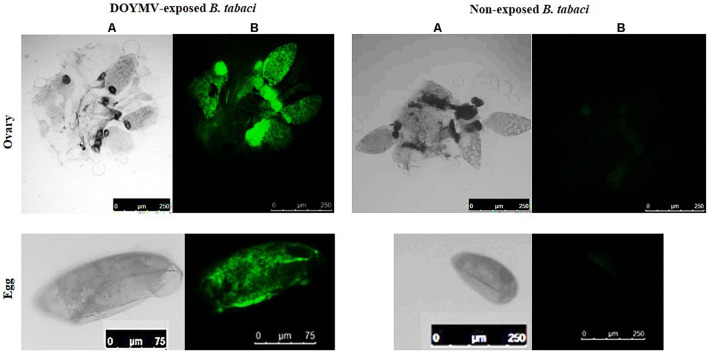
Localization of DoYMV in dissected ovary and eggs of *B. tabaci.* The presence of DoYMV in ovaries (top panel) and eggs (bottom panel) of *B. tabaci* was localized using a Cy3-conjugated nucleic acid probe complementary to the viral strand of DoYMV. **(A)** Bright-field image. **(B)** Cy3-conjugated oligonucleotide probe green fluorescence. Strong Cy3-specific fluorescence was observed in ovaries of *B. tabaci* post 48 h exposure to DoYMV and in the eggs laid on 4 days post DoYMV acquisition by *B. tabaci.* No corresponding fluorescence signal was observed in ovaries and eggs of non-exposed *B. tabaci*.

The presence of DoYMV was localized in ovaries and eggs of *B. tabaci* adult females post virus exposure using a Cy3-probe. The specimens were washed until no viral DNA was detected by PCR from the wash to avoid contaminations from the hemolymph. Strong Cy3-specific fluorescence was observed in ovaries of *B. tabaci* post 48 h of exposure to DoYMV (Figure 3). The presence of fluorescence in ovaries of *B. tabaci* indicated that DoYMV invaded the reproductive system of *B. tabaci*. Localization of DoYMV was also distinguished by Cy3-fluorescence in the eggs laid by the DoYMV-exposed *B. tabaci* parents. The results were consistent with the detection of DoYMV in eggs by PCR and real-time PCR. No corresponding fluorescence signal was observed in ovaries and eggs of aviruliferous *B. tabaci* that indicated the specificity of the assay.

### Propagation of DoYMV in *B. tabaci*

**FIGURE 4:**
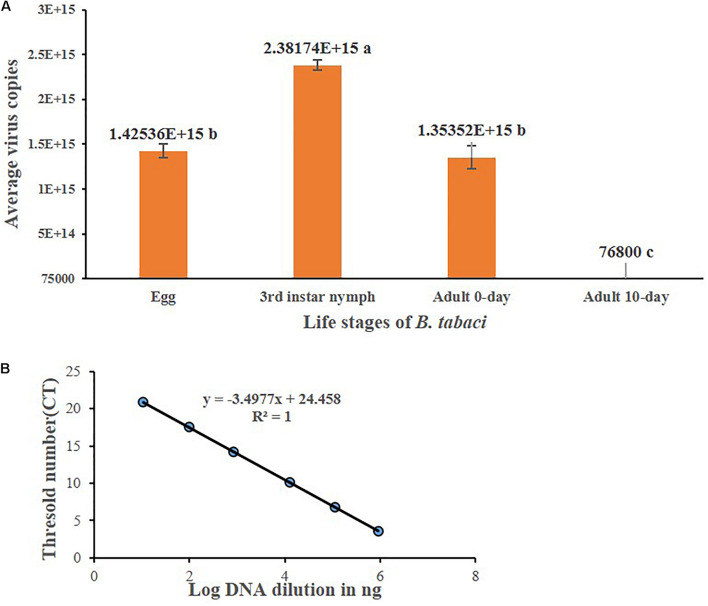
DoYMV copies in different life stages of *B. tabaci.* **(A)** Virus titer was quantified in eggs, third instar nymphs, freshly emerged adults, and 10-day-old adults of F1 progeny. The virus titer increased from egg to nymphal stage. The virus titer dropped down in freshly emerged adults and further reduced in 10-day-old adults. An increase of DoYMV during nymphal instars indicated its propagation in *B. tabaci* for a brief period. The average copy numbers of DoYMV were calculated out of three biological and three technical replicates. Data are means ± SEM, *n* = 36. Errors bars are SEM. Means denoted by different letters indicate a significant difference (*p* < 0.05). **(B)** Standard curves of real-time PCR showed a linear relationship between log DNA concentrations and *C*_*T*_ values for DoYMV. Each concentration was replicated thrice. The equation of the straight line and the coefficient of correlation (*R*^2^) are represented on the graph. The efficiency (E) is 93.15%.

Virus titer was quantified in eggs, third instar nymphs, freshly emerged adults, and 10-day-old adults of F1 progeny. The F1 progeny developed from the eggs laid on 4 days post acquisition was considered for this study. The virus titer started increasing during the nymphal instars. A 1.68-fold increase in the virus titer was recorded in the third instar *B. tabaci* compared with the egg (Figure 4). The virus titer estimated in nanograms using the standard curve in real-time PCR was converted into a copy number profile. The standard curve of DoYMV (Figure 4) showed high amplification efficiency of 93.15% indicating acceptable conditions for absolute quantification. The standard curve showed a coefficient of correlation (*R*^2^) of 1. The amplicon produced a specific peak at 81°C without any secondary peak in melt curve analysis, indicating the specificity of reaction. The eggs laid on 4 days post virus acquisition by *B. tabaci* parents contained on an average 1.43E + 15 copies of DoYMV, whereas it was 2.38E + 15 copies in third instar nymphs. The virus titer dropped down in freshly emerged adults and was almost equivalent to eggs. The virus copies were further reduced to 7.68E + 4 copies in 10-day-old adults. An increase in DoYMV copies during nymphal instars indicated its propagation in *B. tabaci* for a brief period. The propagation might be for a short period and started declining in adult stage.

## Discussion

Dolichos bean is one of the important vegetables grown in Asia and Africa. The cultivation of dolichos has been severely affected by DoYMV. The incidence of DoYMV has increased by 40% post-1990s. Since it was first recorded in 1950 by Capoor and Varma, the etiology, epidemiology, diagnostics, and host plant resistance of DoYMV have been reported by several workers (Yaraguntaiah and Govindu, 1964; Ramakrishnan et al., 1972; Raj et al., 1988; Harrison et al., 1991; Swanson et al., 1992; Maruthi et al., 2005, 2006; Akram et al., 2015). Infected plants produce characteristic yellow mosaic symptoms similar to mungbean yellow mosaic virus (MYMV) and mungbean yellow mosaic India virus (MYMIV), but DoYMV does not infect black gram, cowpea, French bean, green gram, and soybean (Maruthi et al., 2006). Infection at an early stage of crop growth often leads to total yield loss. DNA-A of DoYMV consists of 2,761 nucleotides that indicate the presence of six ORFs typical of Old World begomoviruses (Akram et al., 2015; Suruthi et al., 2018). DoYMV DNA-A shows nearly 63% nucleotide identity with MYMV and MYMIV and lower identities with other begomoviruses (Maruthi et al., 2005). DNA-B consists of 2,733 nucleotides with 57.5–61.0 identity with other legume-infecting begomoviruses (Akram et al., 2015).

Prior to this study, it was thought that DoYMV was transmitted by *B. tabaci* in a persistent-circulative manner like most of the begomoviruses. The preliminary observations during a screening of dolichos germplasms against DoYMV prompted us to investigate the possibilities of transovarial transmission of DoYMV by its vector, *B. tabaci*. In the present study, we have examined if DoYMV is carried from parent to progenies of *B. tabaci* Asia II 1 and propagates in its vector. The rationale of the hypothesis was based on the survival of DoYMV in absence of host crops and bridging the gap between the growing seasons of dolichos. DoYMV has a very narrow host range (Maruthi et al., 2006) and no potential alternate host is known to date that can serve as a reservoir of DoYMV. Symptoms of DoYMV develop almost immediately after germination in open field conditions. Although seed transmission of DoYMV has been reported recently, seed infection does not produce symptoms in plants (Suruthi et al., 2018). We have postulated that *B. tabaci* plays a reservoir for the virus inoculum that is passed vertically to the progenies and immediately infects the germinated plants to produce symptoms.

We have tested the transovarial transmission of DoYMV in progenies of *B. tabaci* by PCR, real-time PCR, Southern blot hybridization, and biological transmission. Eggs laid by DoYMV-exposed *B. tabaci* were found to carry the virus in a unique pattern. DoYMV might take some time to circulate in *B. tabaci* system and invade the reproductive organs. Eggs laid immediately after acquisition were free from DoYMV as tested in PCR and real-time PCR. Only the eggs laid in between 3 and 6 days post virus acquisition by parent *B. tabaci* carried the viral DNA. Eggs laid 7 days post virus acquisition were free from DoYMV again. The absence of DoYMV in eggs beyond this period might be due to the decreasing titer of the virus in the ovaries post acquisition. DoYMV might invade the eggs present in the parent’s ovary during virus acquisition but not infect the germline or is not retained in the ovary for a longer period. Hence, the freshly developing eggs in the ovary post 7 days of the acquisition probably escape the infection. An increase in acquisition exposure further increased the passage of DoYMV in eggs beyond 6 days. Further, the individual eggs from each day laying were tested in real-time PCR to understand the frequency of transovarial transmission. It was interesting to note that not all eggs laid between 3 and 6 days post virus acquisition were infected with DoYMV. About 31–53% of these eggs were DoYMV positive in real-time PCR. The presence of the DoYMV in F1 eggs and ovaries was further substantiated by the hybridization of a Cy3-conjugated nucleic acid probe complementary to 20 nucleotide sequences of DoYMV CP. No viral DNA was detected in PCR from the wash of eggs and ovaries that overruled the possibilities of hemolymph contamination. The developing eggs in parent’s ovary were infected by DoYMV as indicated by the Cy3-labeled fluorescence post 48 h of virus acquisition. The bacteriocyte spheres in the developing eggs in ovary and eggs were distinctly visible under a microscope. Endosymbionts are incorporated during egg maturation and carried to the next generations (Costa et al., 1996). Strong Cy3-specific fluorescence in the bacteriocyte spheres indicated infection of DoYMV. The possible role of bacteriocytes in the transovarial transmission of DoYMV by *B. tabaci* will be worthy of future study to investigate the possible role of bacteriocytes in the transovarial transmission of DoYMV in *B. tabaci*. The presence of TYLCV and TYLCSV DNA in *B. tabaci* MEAM1 and MED eggs was earlier reported by Ghanim et al. (1998), Bosco et al. (2004), and Guo et al. (2019). The specific interaction between TYLCV CP and *B. tabaci* MEAM1 vitellogenin determines the virus entry in *B. tabaci* ovary (Wei et al., 2017). The molecular mechanism by which DoYMV penetrates the reproductive system of *B. tabaci* Asia II 1 may be worth further investigation.

In the present study, viral DNA was also detected in the next-generation adults of *B. tabaci* Asia II 1. About 27–37% of F1 adults generated from the eggs laid between 3 and 6 days post virus acquisition was tested DoYMV positive in real-time PCR. The decrease in the proportion of adults infected with DoYMV than eggs might be due to the reduction of virus titer in the adult stage. Our results are consistent with Bosco et al. (2004) where TYLCSV DNA was detected in eggs but to a lower extent in adults. However, TYLCSV DNA was inherited in F1 adults of *B. tabaci* but not the infectivity. The inherited TYLCSV DNA was unable to give rise to infection (Bosco et al., 2004) and therefore lacks epidemiological significance. On the contrary, the adult progenies of TYLCV-exposed *B. tabaci* MEAM1 were able to infect tomatoes and produce symptoms (Ghanim et al., 2009; Wei et al., 2017). Besides *B. tabaci* MEAM1, neither TYLCV DNA nor the infectivity can be transovarially transmitted by any other cryptic species of *B. tabaci* including Asia II 1 (Ghanim et al., 1998; Guo et al., 2019). In the present study, F1 adults of *B. tabaci* Asia II 1 carrying the DoYMV DNA could also transmit the infectivity to healthy dolichos plants and produced strong typical symptoms. All the tested plants inoculated by F1 adults were tested DoYMV positive in PCR. The distinction of results with previous studies may be due to the virus species and *B. tabaci* cryptic species. Guo et al. (2019) noted the varying ability of *B. tabaci* cryptic species in respect to the transovarial transmission of TYLCV. The transmission of DoYMV from F1 adults to F2 eggs was also evident in the present study that indicated *B. tabaci* Asia II 1 can serve as a reservoir of DoYMV beyond one generation in absence of host plants. During the course of experiment, we also assessed other predominant begomoviruses like MYMIV, tomato leaf curl New Delhi virus (ToLCNDV), and croton yellow vein mosaic virus (CYVMV). No viral DNA of MYMIV, ToLCNDV, and CYVMV was detected in *B. tabaci* progenies.

Begomoviruses are believed not to replicate in *B. tabaci* (Pakkianathan and Ghanim, 2014). DoYMV copies were quantified in different life stages of *B. tabaci* in real-time PCR. An increase in viral copies from egg to nymphal instar indicated propagation of DoYMV in *B. tabaci*. However, the increase was for a short period and decreased thereafter. Replication of DoYMV in *B. tabaci* may be attributable to the evolution of the species under high selection pressure with a very narrow host range. Our results are consistent with Ohnishi et al. (2009), Pakkianathan et al. (2015), and Wang et al. (2016) that reported replication of monopartite TYLCV in *B. tabaci* at the initial level and then decreased. A decrease in begomovirus titer in adults might be due to the transcript accumulation and decay associated with the begomovirus-induced upregulation of the autophagy pathway (Wang et al., 2016). However, two studies concluded that there was no evidence of TYLCV replications in *B. tabaci* (Becker et al., 2015; Sánchez-Campos et al., 2016). The present study is the first evidence of replications of a bipartite begomovirus in *B. tabaci* Asia II 1.

The study provides a pattern of transovarial transmission of DoYMV infectivity by *B. tabaci* Asia II 1 and discusses its implications in disease spread. Seed transmission of DoYMV has also been reported by Suruthi et al. (2018) recently. About 46% of the plants germinated from seeds of infected plants were tested DoYMV positive in DAS ELISA with ToLCNDV polyclonal antibody. However, none of the plants that tested positive in DAS ELISA produced symptoms. The seed transmission as reported may not have much economic significance as the plants did not produce symptoms even after 1 month. The transovarial transmission and replication of DoYMV in its vector *B. tabaci* are therefore important facets of virus–vector interactions and have great epidemiological relevance. In the absence of an alternate host plant, *B. tabaci* serves as a major host of DoYMV to bridge the gap between cropping seasons and are immediately available to infect when dolichos plants are germinated in the field. The present study would help to better understand the virus–vector relationship and disease epidemiology, and formulate strategies for effective management.

## Data Availability Statement

Sequences have been deposited to GenBank under accession numbers MT920041, MZ821026, and MZ503593.

## Author Contributions

AG conceived and designed the research and wrote and edited the manuscript. BR and AG conducted the experiments and recorded the experimental data. AN conducted the dissection and hybridization. SD supplied the dolichos seeds and initial virus inoculum. AG and SM reviewed the data. AD performed the analysis and prepared the figures and tables. SM reviewed the final manuscript. All the authors read and approved the manuscript.

## Conflict of Interest

The authors declare that the research was conducted in the absence of any commercial or financial relationships that could be construed as a potential conflict of interest.

## Publisher’s Note

All claims expressed in this article are solely those of the authors and do not necessarily represent those of their affiliated organizations, or those of the publisher, the editors and the reviewers. Any product that may be evaluated in this article, or claim that may be made by its manufacturer, is not guaranteed or endorsed by the publisher.

## Footnotes

1
www.ncbi.nlm.nih.gov
